# A reference document on *Permissible Limits* for solvents and buffers during *in vitro* antimalarial screening

**DOI:** 10.1038/s41598-018-33226-z

**Published:** 2018-10-08

**Authors:** Renugah Naidu, Gowtham Subramanian, Ying Bena Lim, Chwee Teck Lim, Rajesh Chandramohanadas

**Affiliations:** 1 Pillar of Engineering Product Development (EPD), Singapore University of Technology and Design (SUTD), Singapore, 487372 Singapore; 2 Department of Biomedical Engineering, National University of Singapore, Singapore, 117583 Singapore; 3 Singapore-MIT Alliance for Research and Technology (SMART) Centre, Infectious Diseases IRG, Singapore, 138602 Singapore; 4 Mechanobiology Institute, National University of Singapore, Singapore, 117411 Singapore

## Abstract

Antimalarial drug discovery expands on targeted and phenotype-based screening of potential inhibitory molecules to ascertain overall efficacy, phenotypic characteristics and toxicity, prior to exploring pharmacological optimizations. Candidate inhibitors may have varying chemical properties, thereby requiring specific reconstitution conditions to ensure solubility, stability or bioavailability. Hence, a variety of solvents, buffers, detergents and stabilizers become part of antimalarial efficacy assays, all of which, above certain threshold could interfere with parasite viability, invasion or red blood cell properties leading to misinterpretation of the results. Despite their routine use across malaria research laboratories, there is no documentation on non-toxic range for common constituents including DMSO, glycerol, ethanol and methanol. We herein constructed a compatibility reference guide for 14 such chemicals and estimated their *Permissible Limit* against *P*. *falciparum* asexual stages at which viability and replication of parasites are not compromised. We also demonstrate that at the estimated *Permissible Limit*, red blood cells remain healthy and viable for infection by merozoites. Taken together, this dataset provides a valuable reference tool for the acceptable concentration range for common chemicals during *in vitro* antimalarial tests.

## Introduction

Antimalarial drug discovery relies on high throughput screening of potentially active molecules (or mixtures) against asexual developmental cycle of the malaria parasite. Candidate inhibitors may be chemical (small molecules) or biological (peptides, antibodies or complex extracts) in nature. Hence, the choice of reconstitution conditions to evaluate their inhibitory potential takes into account chemical and biological stability, solubility and storage conditions which introduces a range of solvents, detergents and stabilizers into antimalarial efficacy determination assays. Phosphate-buffered saline (PBS) remains the most popular solvent for a wide-spectrum of chemical and biological agents soluble under aqueous conditions^1^, including dihydroartemesinin^2^. Dimethyl sulfoxide (DMSO) on the other hand, forms a universal solvent for polar and nonpolar compounds alike, ensuring a miscible solution. For instance, large chemical libraries such as the Medicines for Malaria Venture (MMV) Malaria Box^3^, a collection of small molecules on which extensive mode of action research is currently being done^4^, is distributed in 100% DMSO. Apart from PBS and DMSO, other solvents such as ethanol, methanol and acetone, are also used in antimalarial screening. Methanol is also used for reconstituting antimalarial drugs such as artemisinin^5^, mefloquine^6^, quinines and dihydroartemisinin^7,8^.

In addition to solvents, chemicals used as preservatives (such as sodium azide and glycerol)^9^, buffer ingredients and stabilizers (imidazole, urea and glycine)^10–12^, salt^13^ and sugars (trehalose and glucose)^14^ are often part of antimalarial assays. Glycerol is widely used as cryoprotectant for long-term storage of peptides and antibodies^15^. Due to its viscous and hygroscopic nature, glycerol above certain concentrations may affect the ability of malaria parasites to invade red blood cells (RBCs) in culture. It is also possible that glycerol can cause RBC aggregation, in turn affecting parasite invasion. Another commonly utilized preservative is sodium azide, often used in the 0.02–0.05% range^16,17^ due to its bacteriostatic effects^18^.

Glycine and imidazole are used during various steps of protein expression and purification, which often calls for buffer replacement before it is used for functional studies. Glycine methyl ester substituted polyphosphazene conjugates have been tested for *in vitro* release studies of lumefantrine^19^. Urea, a protein denaturant, is used to solubilize large proteins as is capable of disrupting noncovalent bonds in polypeptides and proteins^20^. Cytotoxic effects introduced by such components above certain limits may result in direct inhibition on intracellular parasite viability, replication or reduction in invasion efficiency of merozoites. In addition, chemical or mechanical damage of prospective host RBCs in presence of these chemicals cannot be ruled out. Since, there exists no documentation on the non-toxic range, we created a reference document on the *Permissible Limit* (***PL***) for 14 common chemicals, at which neither *P. falciparum* replication nor host RBCs are affected.

## Materials and Methods

### Blood collection and storage

All experimental procedures were conducted in accordance with approved institutional guidelines of the Singapore University of Technology and Design (SUTD). Blood for plasmodium culture was purchased from Interstate blood bank, USA. Upon collection, blood was centrifuged at 600 × g for 10 minutes, after which buffy coat was removed and the remaining RBCs were washed three times in RPMI 1640 (R8758 Sigma-Aldrich). Washed RBCs were stored in Malaria Culture Media (MCM) at a hematocrit of 50%.

### Preparation of stock and working concentrations of drugs

All chemicals and drugs were purchased from Sigma-Aldrich while PBS was purchased from Invitrogen and glycerol was a product of First base. The range of concentrations of chemicals used in this study are provided in Table 1. All reagents were freshly prepared in the appropriate concentrations in deionized water and filtered using a 0.2 µM filter unit (Sartorius, Singapore). The concentration ranges used in the assays are elaborated in the relevant sections.

**Table 1:** Solvents tested with their corresponding concentration range and estimated *Permissible Limits*.

Chemicals	Concentration range (Units)*	Estimated *Permissible Limit* (*PL*)
**Solvents**		
Dimethyl Sulfoxide (DMSO)	0.0024–5 (%)	0.04%
Phosphate buffered Saline (PBS)	0.0024–5 (x)	0.02x
Ethanol	0.0024–5 (%)	0.16%
Methanol	0.0024–5 (%)	1.25%
**Constituent buffers/stabilizing agents**		
Glycerol	0.0024–5 (%)	1.25%
Acetone	0.0024–5 (%)	0.625%
Acetic acid	0.0024–5 (%)	0.01%
Imidazole	0.0107–22 (mg/ml)	0.02 mg/ml
Urea	0.00048–1 (mg/ml)	0.24mg/ml
Glycine	0.0298–61 (mg/ml)	0.31mg/ml
Sodium azide	0.0012–2.5 (mg/ml)	0.001 mg/ml
**Salt and Sugars**		
Sodium chloride	0.0256–52.5 (mg/ml)	0.21 mg/ml
Glucose	0.0261–53.5 (mg/ml)	1.67 mg/ml
Trehalose	0.0249–51 (mg/ml)	0.16 mg/ml

### *P. falciparum* lines and culture methods

3D7 line of *P. falciparum* was used for all experiments. Parasites were cultured at 2.5% hematocrit in Malaria Culture Medium (MCM): RPMI-HEPES supplemented with hypoxanthine (50 μg mL^−1^, Life technologies), NaHCO_3_ (25 mM), gentamicin (2.5 μg mL^−1^, Life technologies), and Albumax II (0.5% wt/vol, Life technologies). To obtain synchronous parasites, schizont stage parasites (46–48 hpi) were collected through magnetic-activated cell sorting (MACS, Miltenyi Biotec, Singapore) and incubated with fresh RBCs for 3 hours, followed by treatment with 5% sorbitol (Sigma-Aldrich) to select ring-stage infections.

### Parasitemia determination through flow cytometry analysis

Flow cytometry was carried out to quantify and categorize specific parasite stages: rings, trophozoites and schizonts based on DNA content using an Accuri C6 (BD Biosciences) flow cytometer. A minimum of 100,000 events were recorded for each sample. To determine parasitemia, 100 μl aliquots of culture was fixed with 0.1% glutaraldehyde (Sigma-Aldrich) at 4 °C overnight. Cells were washed in PBS and permeabilized using 0.25% Triton X-100/PBS (Sigma-Aldrich) for 10 minutes at room temperature. After washing, samples were incubated with 25 μg/ml Hoechst 33342 (Thermo Fisher) for 30 minutes in dark and quantified using flow cytometry as previously reported^21^. Data analysis and statistics were performed using GraphPad Prism 7 (GraphPad Software).

### Microscopic examination of Giemsa smears

Thin blood smears of *P. falciparum* cultures were prepared on glass slides, fixed with 100% methanol (Merck) and stained with fresh 1:10 Giemsa (Merck) solution made in deionized water. Smears were examined under 100X oil immersion objective microscope (Leica ICC50 W). Images from the smears were captured using a Leica digital camera.

### Micropipette aspiration

A borosilicate glass micropipette was used to aspirate the RBC membrane to determine membrane shear modulus through micropipette aspiration technique. Pipettes were drawn from borosilicate glass tubing (Sutter Instrument Model P-2000) and cut (Narishige MF-900) prior to mounting to the micromanipulator. The micropipette’s inner diameter was approximately 1 ± 0.25 µm. A pressure-drop rate of 1 Pa/s and a total pressure drop of 100 Pa were applied to aspirate and deform each cell. For each treated sample and control sample, a total of 20 cells were measured and analyzed accordingly. The aspiration was visualized on an Olympus IX71 microscope and processed by QCapture Pro 6.0. The maximum time taken for measurement of each sample was approximately 2 hours before replenishing with fresh sample. The recorded aspiration values were manually extracted and the shear modulus was calculated using the Hochmuth model^22^.

## Results and Discussion

### Estimation of *Permissible Limits (PL*) of 14 chemicals in *P. falciparum* assays

We performed literature survey to identify chemicals that routinely appear in antimalarial screening assays, of which, 14 were prioritized for subsequent experiments. The range of concentrations for testing was determined based on literature and summarized in Table 1. For evaluating the inhibitory potential, synchronous cultures of trophozoite stage parasites (24–26 hpi) at 1% parasitemia in 96 well format were incubated with the chemicals. Untreated iRBCs were included as blank controls for all experiments. Samples were harvested in the next cycle (after 50–52 hpi) for microscopic examination and flow cytometry as reported previously^21^. Data analysis and statistical tests were performed using GraphPad Prism 7 (GraphPad Software Inc, USA) to generate a compatibility reference dataset.

Table 1 summarizes the results for the 14 chemicals tested, presented as the mean of three independent experiments performed in duplicates. Dose-dependent effect on parasitemia for all chemicals are individually presented as Supplementary Fig. S1. From these analyses, we determined the (***PL)***, which is defined as the highest concentration of a chemical that ensures 100% parasite survival in comparison to untreated healthy controls. In parallel, we have also carefully examined Geimsa-stained blood smears from each treatment under a microscope. All chemicals at their ***PL*** values appeared to support normal growth and replication of intracellular parasites as well as maintained healthy uninfected RBCs in culture with no lysis, aggregation or morphological changes.

A close look at the ***PL*** values provided interesting observations. For example, highest concentration of DMSO that does not affect parasite proliferation was estimated as 0.0390% while ~50% reduction in parasitemia was recorded at 1% final concentration of DMSO. This is in contrast to its normal inclusion as negative control at 0.5–1% range for routine drug efficacy screens, which can introduce changes in osmolarity and shrinkage of cells^23^. Glycerol was compatible with the assay up to a concentration of 1.25%. In hindsight, storage of antibodies and peptides often involves the inclusion of glycerol up to 20%^24^, making even a 10-fold final dilution above the ***PL*** range we have determined. For sodium azide, which is often used at final concentration of ~0.05%, 10% parasite death was observed in comparison to the ***PL*** value of 0.0001% (1.2207 μg/ml) at which 100% parasites survived.

### Egress and invasion are unaffected at the determined *PLs*

Having determined the compatibility against overall parasite development, we estimated the influence of these chemicals during transition of schizonts to rings. Firstly, the iRBC membrane is known to undergo modifications and become increasingly permeable^25^ during schizogony, making them more susceptible to damage at this stage. Secondly, extracellular merozoites (released during iRBC cytolysis) are no longer protected by the parasitophorous vacuole and RBC membrane. This may expose them to chemicals to a larger extent reducing their invasion potency, considering the short time window for merozoite invasion^26^. Merozoite release and invasion being a dynamic event^27^, changes in ionic balance (salts, sugars and constituents of buffers), physical properties (viscous medium such as glycerol and sugars) or intrinsic toxicity (sodium azide) may also influence the assay.

**Figure 1:**
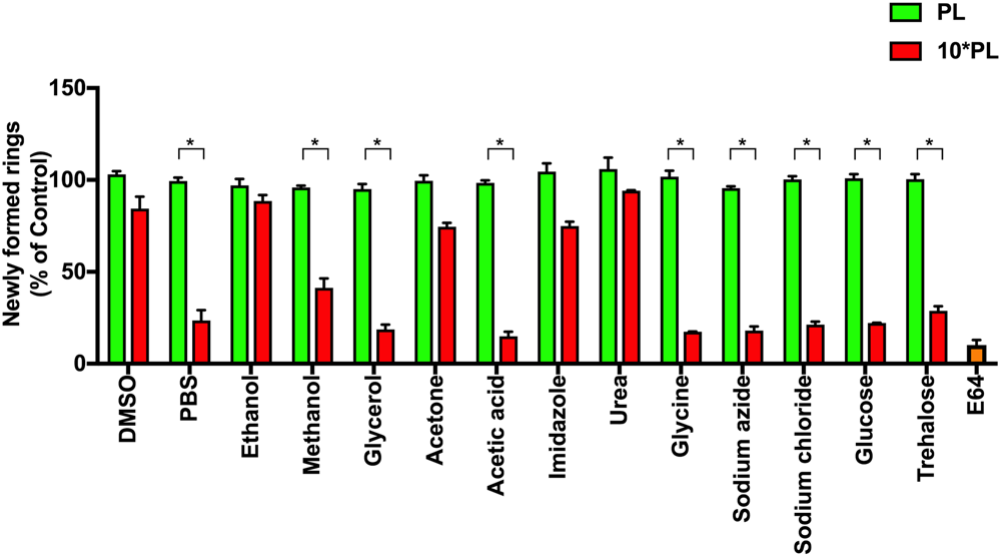
*Permissible Limits* (***PL***) identified support normal replication. Shizont-stage parasites (~42 hpi) incubated at ***PL*** and 10*PL were allowed to egress and re-invade and representative samples were analysed through flow cytometry and microscopy. None of the chemicals were inhibitory to parasite egress or invasion at the ***PL*** compared to E64 (*trans*-Epoxysuccinyl-L-leucylamido(4-guanidino) butane) which served as a positive control for egress inhibition. Bar graphs represent the mean ring-stage populations measured by flow cytometry from three independent experiments (in duplicates) expressed as a percentage of non- treated control population. Error bars represent the standard errors of the means. Significant results are indicated as follows: *<0.001. All other comparisons show no significant differences (Holm-Sidak method, with alpha = 5.000%).

To investigate this, we initially attempted 2*PL, 5*PL, 10*PL and 20*PL, however, significant change in parasite stage-transition was not observed for the first two conditions. Inclusion of 20*PL was either not compatible with the assay (lysed or aggregated RBCs) or obviously toxic to parasites for most chemicals and therefore not pursued. Hence, we chose to introduce all chemicals at ***PL*** and at 10*PL with magnet purified schizont stage parasites (40–42 hpi) at 1% parasitemia. This was followed by parasitemia estimation after 12 hours (Fig. 1), when new ring-stage infections were established. All treatments at ***PL*** resulted in invasion comparable to untreated controls. Although most of the chemicals at 10*PL hindered schizont to ring transition of the parasites to some degree; treatment with urea, imidazole, acetone, ethanol and DMSO did not result in significant reduction in ring formation. In excess of 80% reduction in rings was observed in cultures treated with glycerol, acetic acid, glycine and sodium azide at 10*PL, whereas ethanol and urea showed only 15% reduction of ring population even at 10*PL (Fig. 1). These experiments collectively allowed us to confirm that ***PL*** values are compatible to parasite viability, development as well as transition from schizonts to rings.

### RBC integrity and mechanical properties do not alter at the *PL* range

**Figure 2:**
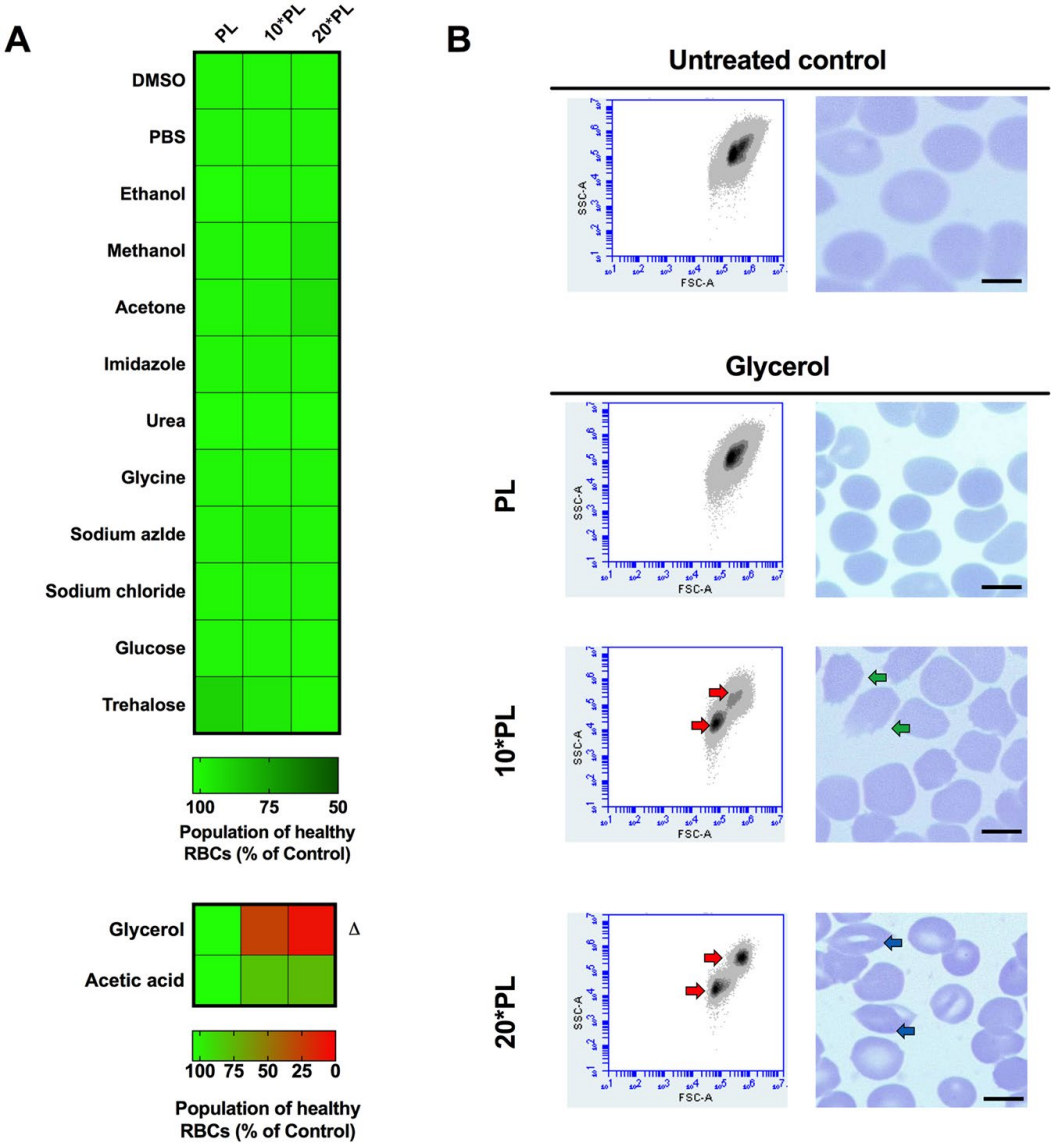
Effect of solvent treatment on the morphology and integrity of RBCs. (**A**) Fresh healthy RBCs (not infected with plasmodium) were treated at ***PL***, 10*PL and 20*PL of all reagents for 48 hours, followed by flow cytometry analysis to examine likely morphological differences. The population of healthy RBCs were comparable to untreated control at the ***PL*** for all chemicals whereas glycerol and acetic acid showed a decrease in RBC population at 10*PL and 20*PL. ∆: There were significant population-wide differences when RBCs were treated with glycerol at ***PL*** and 10*PL and also between ***PL*** and 20*PL (One way Anova with Holm-Sidak’s multiple comparisons test, **0.001 to 0.01). (**B**) Representative flow cytomtery plots and Giemsa-stained images of healthy RBCs treated at ***PL***, 10*PL and 20*PL with glycerol showing an obvious shift (red arrowheads) in RBC population at concentrations higher than ***PL*** likely due to expulsion of RBC contents (green arrowheads) and rhomboid elongated structural change in RBC morphology (blue arrowheads) as observed through microscopy (100X oil magnification Leica Microscope ICC50 W).

Chemical damage leading to RBC fragility, aggregation, hemoglobin conformational change or membrane stiffening may alter their ability to support and sustain plasmodium infection. Although RBCs are capable of withstanding chemical and mechanical stress without undergoing hemolysis, the likely effects caused by chemicals can be a factor on their ability to support continued infection in antimalarial assays, which sometime lasts more than 48 hours. Therefore, we set out to ensure that RBC integrity and properties are not compromised at ***PL*** values for each of the chemicals. To do so, healthy RBCs were pretreated at ***PL***, 10*PL and 20*PL with each of the 14 chemicals. 48 hour post incubation, the RBC pellet was subjected to flow cytometry analysis and whole blood cell population was gated for comparison. The gating was performed based on untreated whole RBC population (Fig. 2B; flow cytometry plot) and was kept consistent for all samples. RBCs treated at ***PL*** were comparable to untreated RBCs in overall distribution (Fig. 2A - top panel), however, 10*PL and 20*PL of glycerol caused significant shift in the population (Fig. 2A - bottom panel; red arrowheads in Fig. 2B) suggesting possible red cell content extrusion and cell deformation. Microscopic inspection of Giemsa-stained images confirmed that RBC contents are extruded at 10*PL of glycerol (green arrowheads in Fig. 2B). Erythrocyte morphology was observed at 20*PL, where RBCs lacked their biconcave discoid structure, but appeared rhomboid and elongated (blue arrowheads in Fig. 2B).

**Figure 3:**
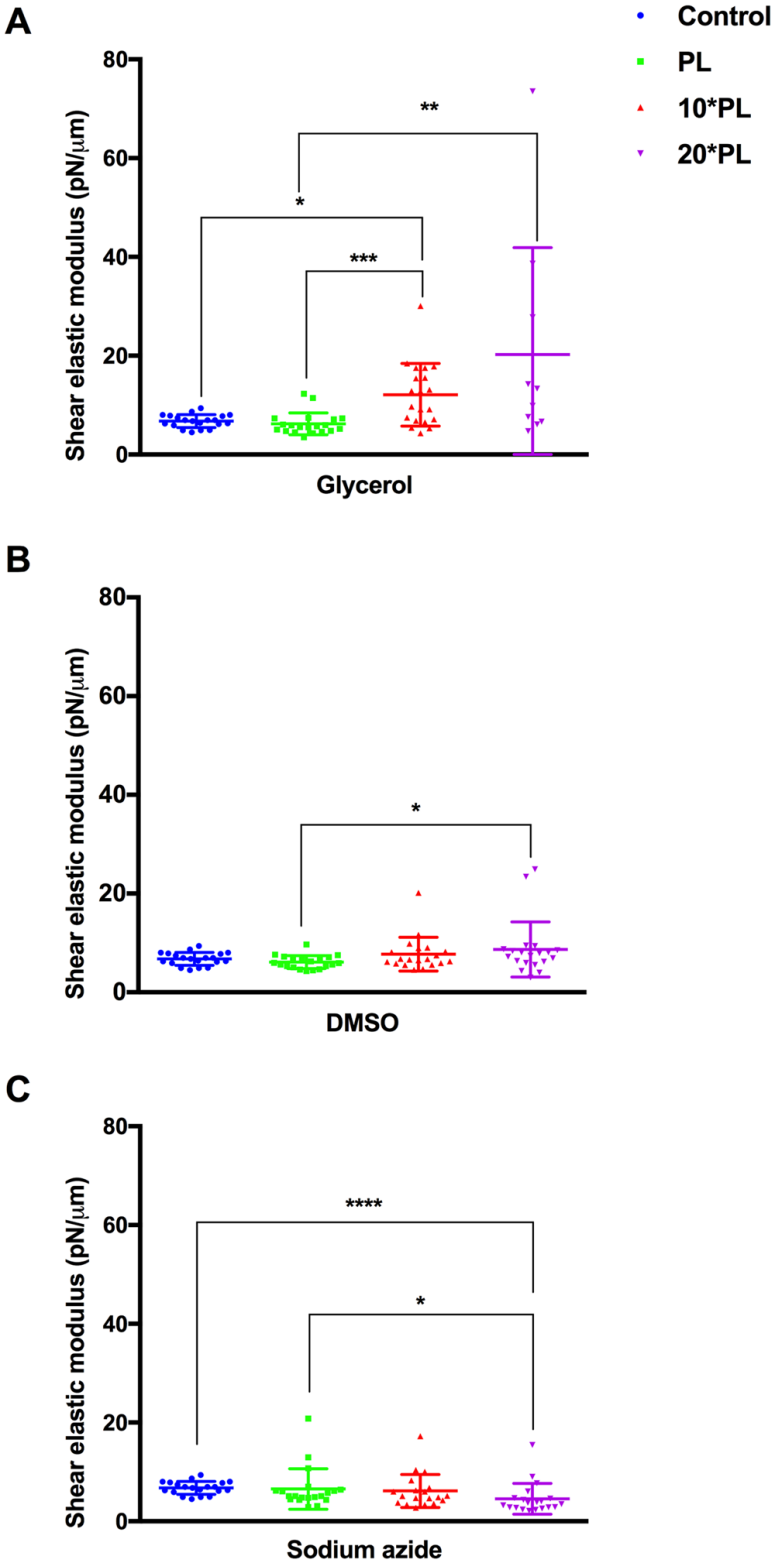
Mechanical properties of healthy RBCs at ***PL*** values for DMSO, glycerol and sodium azide: Healthy RBCs were treated at ***PL***, 10*PL and 20*PL values of (**A**) Glycerol, (**B**) DMSO and (**C**) Sodium azide and membrane shear modulus determined through micropipette aspiration technique. A total sample size of at least 20 cells per measurement was taken for individual experiments. The cell membrane was monitored by Olympus IX71 microscope and processed by QCapture Pro 6.0. The maximum time span before the whole sample was replaced by a fresh sample was 2 hours. From the high-resolution recordings, the leading edge of the aspired RBC membrane was tracked manually for calculating the elastic shear modulus using the Hochmuth model. All RBCs treated at ***PL*** value showed no significant changes in the membrane deformability and were observed to have comparable shear modulus to control population (untreated control). Significant results are indicated as follows: ****<0.0001; ***<0.001; **0.001 to 0.01; *0.01 to 0.05. All other comparisons show no significant differences (Kruskal-Wallis test with Dunn’s multiple comparison test).

As RBC deformability changes arising from chemical damage can reduce malaria infection^28^, membrane stiffness parameters of RBCs exposed at ***PL*** were examined through micropipette aspiration technique. Only three representative chemicals were selected: (**A**) glycerol, (**B**) DMSO and (**C**) sodium azide, owing to low throughput nature of the micropipette aspiration assays. Healthy RBCs were treated at ***PL***, 10*PL and 20*PL for 48 hours (to replicate a typical antimalarial screening assay) under standard culture conditions. A minimum of 20 cells (randomly chosen to avoid possible biases) was measured for each sample and each concentration respectively. The length of the cell aspirated into the micropipette as well as the inner diameter of the micropipette were measured and values were fitted into the model developed by Chien and colleagues^29^. The untreated controls measured an estimated shear modulus (***μ***) between 4–8 pN/μM, as established for healthy RBCs^30^. For the three chemicals tested, treatment at ***PL*** values did not alter the membrane shear modulus compared to untreated samples (Fig. 3A–C). Average shear moduli of 6.11 pN/μM, 6.22 pN/μM and 6.55 pN/μM were recorded for DMSO, glycerol and sodium azide at the ***PL*** values respectively, comparable to untreated control (6.76 pN/μM). There was a considerable increase in shear modulus when RBCs were treated with glycerol at 10*PL (12.10 pN/μM) and 20*PL (20.25 pN/μM), which could be a cause for decrease in parasite densities observed in Fig. 1. Interestingly, RBCs treated at 10*PL and 20*PL resulted in significant increase in viscosity and adhesive properties. In samples treated at high concentrations (10*PL and 20*PL), a modest increase in the membrane stiffness was observed, which were clear outliers, but not omitted from analyses to display the vast variability in measurements.

### Influence of reconstitution conditions on antimalarial efficacy determination

To evaluate the relevance of ***PL*** on overall inhibitory potential determination, we screened *P. falciparum* against 5 antimalarials: (i) chloroquine (ii) artemisinin, (iii) dihydroartemisinin (iv) atovaquone and (v) halofantrine under 6 solubilizing conditions (***PL*** and 10*PL). To do this, solubilizing conditions were chosen based on the drug’s compatibility. Atovaquone (in DMSO), dihydroartemisinin (in (a) ethanol and (b) methanol), halofantrine (in ethanol), chloroquine (in acetic acid) and artemisinin (in acetone) were dissolved at ***PL*** and 10*PL of respective solvents. Similarly, an antibody against human basigin that blocks invasion^31^, was also tested at ***PL*** and 10*PL values of glycerol and sodium azide.

**Figure 4:**
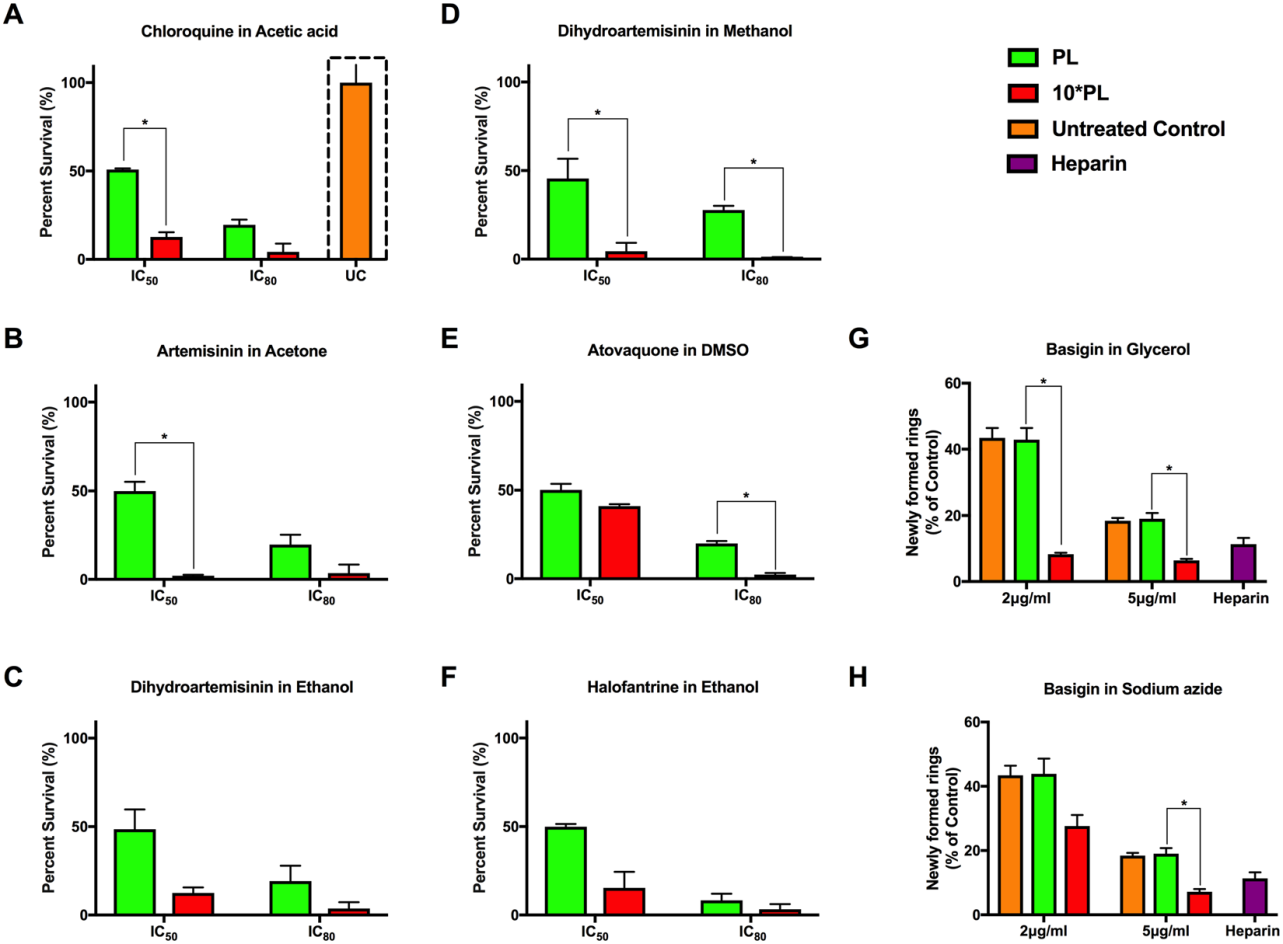
Efficacy determination of selected antimalarials under specific (***PL*** and 10*PL) solvent conditions. Five well-known antimalarials under 6 reconstitution conditions were tested at their documented IC_50_ and IC_80_ values under ***PL*** and 10*PL as below; (**A**) Chloroquine in acetic acid, (**B**) Artemisinin in acetone, (**C**) Dihydroartemisinin in ethanol, (**D**) Dihydroartemisinin in methanol, (**E**) Atovaquone in DMSO and (**F**) Halofantrine in ethanol. Trophozoite stage parasites were maintained with different drugs for 48 hours, followed by parasitemia determination through flow cytometry. Solubilizing conditions above ***PL*** resulted in significantly reduced parasitemia at both IC_50_ and IC_80_ doses, indicating the effect of solvents on parasite viability besides drug-induced killing. The effect of (**G**) glycerol and (**H**) sodium azide above ***PL*** values on parasites was determined by performing invasion assays in presence of anti-basigin antibody (2 μg and 5 μg/ml) in ***PL*** and 10*PL along with heparin as a positive control (at 100 μg/ml). Parasitemia was reduced in presence of 10*PL for both glycerol and sodium azide compared to controls (treated at ***PL*** values or untreated samples). Significant results are indicated as follows: *<0.001. All other comparisons show no significant differences (Holm-Sidak method, with alpha = 5.000%).

Early trophozoite stage parasites (24–26 hpi) at 1% initial parasitemia were exposed to the drugs at their IC_50_ and IC_80_ concentrations as previously established by Wilson and colleagues^32^. Once late trophozoites stage parasites (32–34 hpi) appeared in the next cycle, samples were collected and parasitemia was scored using flow cytometry^33^ for all samples. At ***PL*** values, all drugs reproduced previously established IC_50_ with 50% reduction in parasites as expected. However, presence of solvents at 10*PL resulted in higher parasite death in all cases (Fig. 4A–F). For example, when chloroquine was dissolved in acetic acid at 10*PL, more than ~90% parasites were killed, compared to 50% at ***PL*** values (Fig. 4A). The trend remained comparable for acetone (Fig. 4B, ~90% death at IC_50_ of astemisinin) and ethanol (Fig. 4C, ~87% for DHA and Fig. 4F, ~84% for halofantrine) at respective IC_50_ doses. The effect of DMSO at 10* ***PL*** at IC_50_ level exposure of Atovaquone caused marginal damage (Fig. 4E), yet significant with more than ~58% parasite death instead of 50% in controls.

In the case of anti-basigin antibody, treatment at 2 μg/ml at ***PL*** concentration of glycerol resulted in ~43% parasite invasion which was reduced to only ~8% invasion at 10*PL (Fig. 4G). We observed a similar trend when a higher concentration (5 μg/ml) of the antibody was used, with less than ~6% ring populations detected against ~19% in samples treated at ***PL*** values. A similar experiment was performed by including sodium azide at ***PL*** and 10*PL together with anti-basigin antibody (Fig. 4H). In this case, inclusion of 10*PL of sodium azide in the assay resulted in roughly ~27% invasion in comparison to ~43% at ***PL*** value for 2 μg/ml of anti-basigin and ~7% invasion in comparison to ~19% invasion at 10* ***PL*** at 5 μg/ml. Taken together, these observations clearly indicate the influence of glycerol and sodium azide concentrations on antimalarial invasion assays.

Collectively, drugs dissolved at 10*PL of the solvents led to significant reduction in parasite growth, suggesting the solvent concentrations at which these drugs are dissolved can influence the assay outcome. Evidently, the higher inhibitory potential for drugs were due to the interference caused by the diluents when used at amounts higher than ***PL***. This may be particularly important when analogues of the same molecule are tested in parallel to investigate structure-activity relationships, or when chemical properties of analogues require different solubilizing conditions. At this outset, our results provide a previously undetermined dataset on drug reconstitution conditions at which both the red cell integrity and *P. falciparum* growth and proliferation are not compromised.

## Conclusions

We provide documentation of *Permissible Limits* for 14 commonly used chemicals that are often used to reconsitute drugs and molecules in antimalarial assays. We also demonstrate that the range determined is compatible with various *in vitro* screens without affecting viability or integrity of parasites or host red blood cells.

## Electronic supplementary material

Supplemetary Figure 1

## Data Availability

The datasets used and/or analysed during the current study are available from the corresponding author on reasonable request.
