# Supplementary material for: A reference document on Permissible Limits for solvents and buffers during in vitro antimalarial screening
Source: Sci Rep. 2018 Oct 8;8:14974. doi: 10.1038/s41598-018-33226-z (PMC6175914; doi:10.1038/s41598-018-33226-z)
Supplement: Supplementary file 1 — Supplemetary Figure 1 [file 41598_2018_33226_MOESM1_ESM.pdf]

A reference document on permissible limits for solvents and buffers during *in vitro* antimalarial screening

Renugah Naidu[#1], Gowtham Subramanian[#1], Ying Bena Lim[2,3], Chwee Teck Lim[2,3,4] and Rajesh Chandramohanadas[1*]

[1]Pillar of Engineering Product Development (EPD), Singapore University of Technology and Design (SUTD), Singapore.

[2]Department of Biomedical Engineering, National University of Singapore, Singapore, 117583, Singapore.

[3]Singapore-MIT Alliance for Research and Technology (SMART) Centre, Infectious Diseases IRG, Singapore, 138602, Singapore.

[4]Mechanobiology Institute, National University of Singapore, Singapore, 117411, Singapore.

[#] These authors contributed equally to this work.

[*] Corresponding Author: Rajesh Chandramohanadas Email: rajesh@sutd.edu.sg

Supplementary Figure. 1

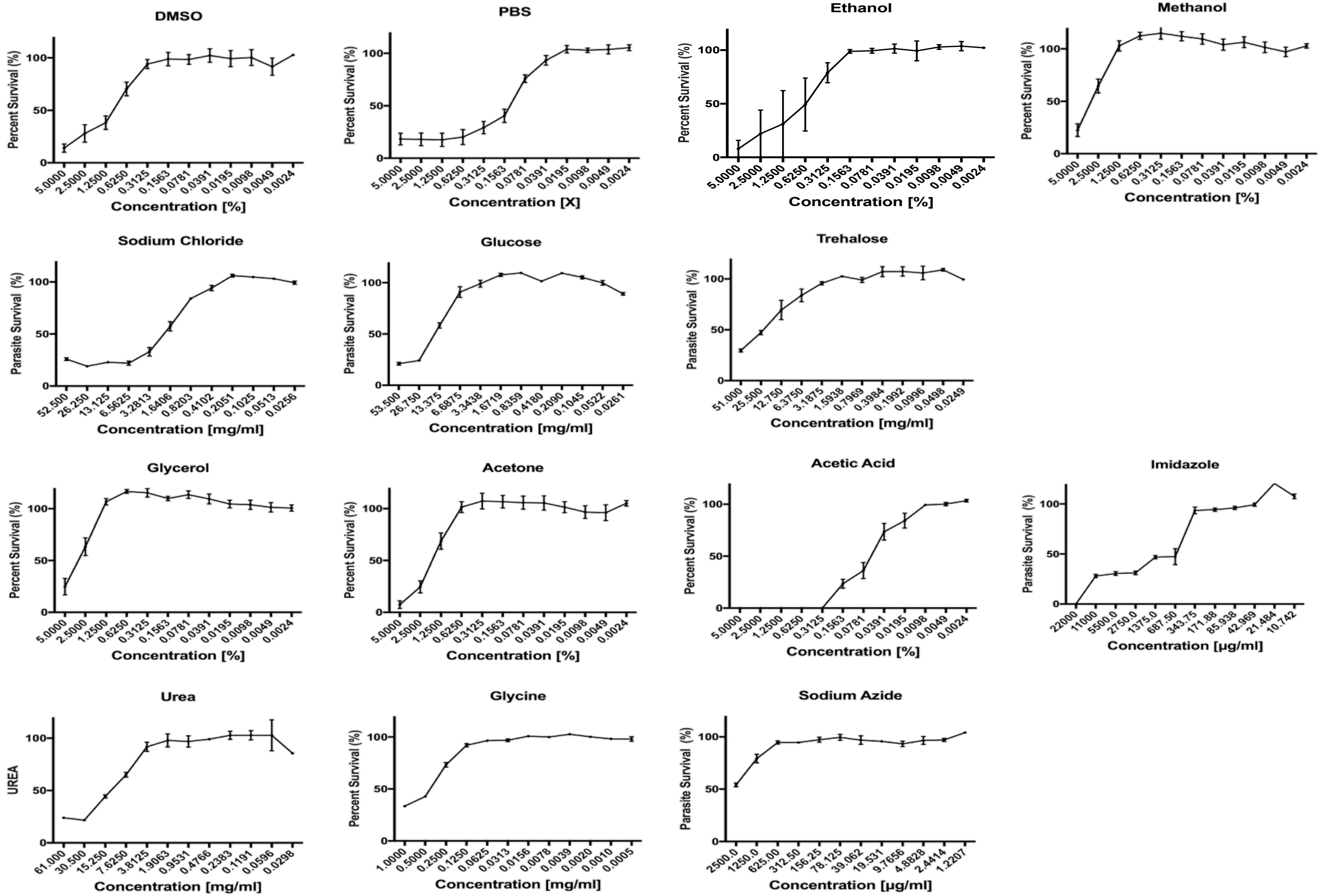

Supplementary Figure S1. **Dose-response curve for individual chemicals.** Briefly, trophozoite stage parasites (24 - 26 h) at 1% parasitemia and 2.5% hematocrit were incubated with 1 in 2-fold serial dilutions of 14 selected chemicals. Samples were maintained undisturbed for 54 to 56 h (until late trophozoites in the next replication cycle), fixed in 0.1% glutaraldehyde and parasitemia scored by flow cytometry. Data in this table represent the means of three (in duplicate) independent experiments.
